# On the value of advanced information about delayed rewards

**DOI:** 10.1007/s10071-024-01856-8

**Published:** 2024-03-02

**Authors:** Alejandro Macías, Armando Machado, Marco Vasconcelos

**Affiliations:** 1 William James Center for Research, University of Aveiro, Aveiro, Portugal; 2 Animal Learning and Behavior Lab, School of Psychology, University of Minho, Campus de Gualtar, 4710-057 Braga, Portugal

**Keywords:** Delay to reward, Information bias, Instrumental value, Pigeons, *Δ*–*Σ* hypothesis

## Abstract

In a variety of laboratory preparations, several animal species prefer signaled over unsignaled outcomes. Here we examine whether pigeons prefer options that signal the delay to reward over options that do not and how this preference changes with the ratio of the delays. We offered pigeons repeated choices between two alternatives leading to a short or a long delay to reward. For one alternative (*informative*), the short and long delays were reliably signaled by different stimuli (e.g., *S*^S^ for short delays, *S*^L^ for long delays). For the other (*non-informative*), the delays were not reliably signaled by the stimuli presented (*S*^1^ and *S*^2^). Across conditions, we varied the durations of the short and long delays, hence their ratio, while keeping the average delay to reward constant. Pigeons preferred the informative over the non-informative option and this preference became stronger as the ratio of the long to the short delay increased. A modified version of the *Δ*–*Σ* hypothesis (González et al., J Exp Anal Behav 113(3):591–608. https://doi.org/10.1002/jeab.595, 2020a) incorporating a contrast-like process between the immediacies to reward signaled by each stimulus accounted well for our findings. Functionally, we argue that a preference for signaled delays hinges on the potential instrumental advantage typically conveyed by information.

## Introduction

**Fig. 1:**
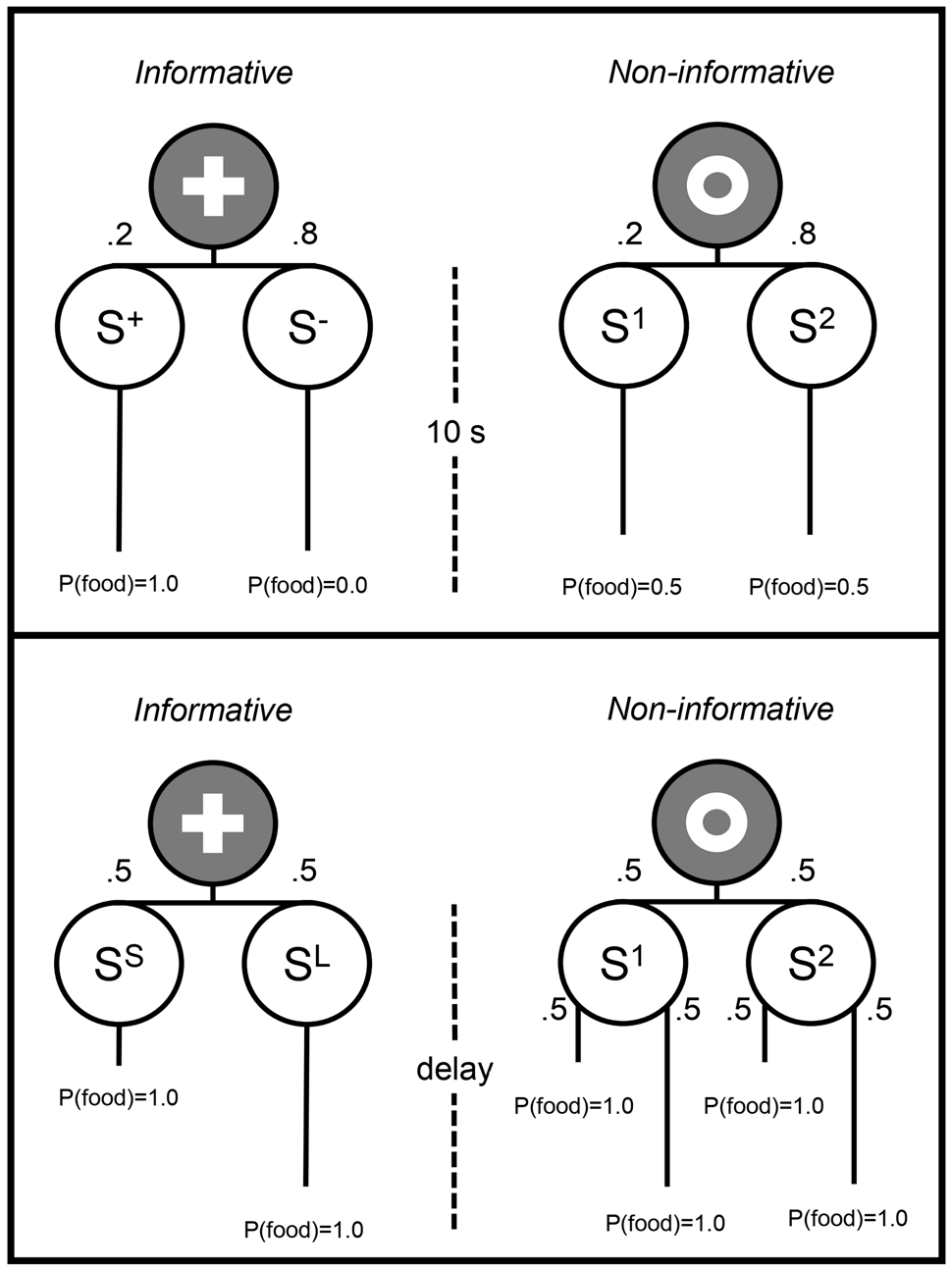
Suboptimal and delay-based choice tasks. Note. Pigeons choose between an informative (cross) and a non-informative (circle) option. Top panel: Once a choice is made, animals see one of two possible 10-s long terminal stimuli per alternative. Each stimulus occurs 20% (*S*^+^ and *S*^1^) or 80% of the time (*S*^−^ and *S*^2^). *S*^+^ is always reinforced, *S*^−^ is never reinforced, and S^1^ and S^2^ are reinforced with a 0.5 probability. Bottom panel: Both options are followed either by a short or a long delay to food, each 50% of the time, and are always reinforced. In the informative option, the *short* and *long* delays are signaled by *S*^S^ and *S*^L^, respectively. In the non-informative option, the delays are equally likely in the presence of *S*^1^ and *S*^2^ (i.e., they are not signaled)

Under some conditions, animals engage in an unprofitable bargain: While hungry, they trade unusable information for food by preferring an informative but suboptimal option to a non-informative but optimal one. The typical procedure, known as the ‘suboptimal choice task’, is depicted in the top panel of Fig. 1. The animal faces repeated choices between the two options. If it chooses the informative option (cross in the figure), on 20% of the occasions an S^+^ stimulus is presented, and 10-s later food is delivered; in the remaining 80% of the occasions an S^−^ stimulus is presented, and 10 s later no food is delivered. If the animal chooses the non-informative option instead (circle in the figure), stimulus *S*^1^ or *S*^2^ follows for 10 s and then food occurs on 50% of the occasions regardless of the stimulus shown. In other words, choice is between an option that signals the trial outcome, but the outcome consists mostly of no food and rarely of a bit of food, and another option that does not signal the outcome, but the outcome consists of significantly more food. Although the non-informative option usually yields more reward (2.5 times more in the figure), human gamblers (Molet et al. 2012), pigeons (e.g., Fortes et al. 2018; Gipson et al. 2009; Smith et al. 2016; Stagner & Zentall 2010), starlings (Vasconcelos et al. 2015), rhesus macaques (Blanchard et al. 2015), and rats (e.g., Ajuwon et al. 2023; Chow et al. 2017; Cunningham and Shahan 2019, 2020) prefer the informative option.

This seemingly paradoxical preference generated an intense empirical and theoretical effort to understand its causes (e.g., Cunningham and Shahan 2018; Daniels and Sanabria 2018; Dunn et al. 2023; González et al. 2020a; McDevitt et al. 2016; Vasconcelos et al. 2018; Zentall 2016). Whatever the mechanism involved, this preference for informative signals when they do not yield any tangible instrumental benefit is reminiscent of the information-seeking hypothesis first suggested in the ‘observing response’ literature. In the original preparation, Wyckoff (1969) presented pigeons with a white key where a mixture of two equiprobable, non-signaled reinforcement schedules alternated unpredictably. One offered food every 30 s (FI 30 s) and the other never produced food (extinction). Importantly, if the pigeon stepped on a pedal, then the white key turned green when the FI 30 s schedule was in effect and red when extinction was in effect. In other words, stepping on the pedal provided information about the state of the world and was labeled the ‘observing response.’ Even though the information could not be used to change the outcome (food or no food), pigeons readily learned to press the pedal (see also Browne & Dinsmoor 1974; Dinsmoor et al. 1972; Mulvaney et al. 1974; Prokasy 1956; for a review, see Dinsmoor 1983). In a related paradigm, rats prefer alternatives wherein forewarning information about impending unavoidable shock is given over alternatives with no such information (e.g., Lockard 1963; Perkins et al. 1963).

The preceding examples suggest that animals may be biased to perform responses that reduce the uncertainty about future events, despite being unable to change the occurrence of these events. If this is a somewhat general proclivity, then it should extend to other biologically relevant situations beyond the presence or absence of food or shock. This bias should emerge in any preparation in which a response produces reliable information about an upcoming biologically relevant event, whether it regards its quantity, delay, probability, or quality.

Bromberg-Martin and Hikosaka (2009; see also Bromberg-Martin and Hikosaka 2011) tackled the issue of magnitude. They modified the suboptimal choice task (cf. top panel of Fig. 1) to study the effect of signaling the magnitude of impending rewards. Using a preparation where both the informative and non-informative options led to a small or large reward with equal probability (i.e., the choices did not affect the rate of reward), they found that, all else equal, macaque monkeys prefer options that signal the size of the upcoming reward over options that do not (see also Laude et al. 2014; Zentall and Stagner 2011).

This study focuses on another dimension of reward—the delay to collect it. Suppose that animals face repeated choices between two options, one informative, the other non-informative. Both options lead to reward, half of the trials after a short delay and the other half after a long delay (see bottom panel of Fig. 1). The difference between the options is that, in the informative option, the delays are perfectly correlated with the terminal stimuli (e.g., Green during the short delay, *S*^S^, and Red during the long delay, *S*^L^), whereas in the non-informative option, the delays are not correlated with such stimuli (e.g., Yellow, *S*^1^, and Blue, *S*^2^, during S^S^ and *S*^L^). The two options yield the same rate of reward, and choices do not affect the obtained rate. Will animals prefer signaled over unsignaled delays to reward under these conditions?

A modified version of the *Δ*–*Σ* hypothesis provides a prediction. Developed to account for the standard result in the suboptimal choice task, the hypothesis assumes that the value of an alternative depends on two high-order variables: the difference between the two reinforcement probabilities associated with the terminal-link stimuli of each option, ∆, and the overall probability of reinforcement of an option, ∑ (cf. top panel of Fig. 1; see González et al. 2020a, b for further details). A modified version of the *Δ*–*Σ* hypothesis suggests that animals should not only prefer signaled to unsignaled delays but also that the strength of preference should depend on the disparity between possible delays. To see why, consider first the suboptimal choice task (top panel of Fig. 1). According to the model, the value of an alternative depends on two higher-order variables: (1) Delta (*∆*), the difference between the reward probabilities associated with each stimulus within each option (a contrast-like effect), and (2) Sigma (∑), the overall probability of reward associated with each option. These two variables determine the overall value of an option according to the equation

1 $${V}_{i}={\left({\Sigma }_{i}\right)}^{c}*{e}^{\beta *{\Delta }_{i}},$$

with the scaling parameters *c* and *β* both > 0, and *i* standing for the “info*”* or “noninfo*”* option. Preference for the informative option can be estimated by Luce’s ratio (1959), *V*_info_/(*V*_info_ + *V*_noninfo_), which simplifies to

2 $${P}_{info} =\frac{1}{1+{\left(\frac{{\Sigma }_{{\text{noninfo}}}}{{\Sigma }_{{\text{info}}}}\right)}^{c}{e}^{-\beta \left({\Delta }_{{\text{info}}}-{\Delta }_{{\text{noninfo}}}\right)}}$$

Equation 2 predicts indifference between the options in the delay-based version of the task (cf. bottom panel of Fig. 1). This is because the probability of reward with each terminal stimulus is 1.0 (thus, both *Δ*_info_ and *Δ*_noninfo_ are zero) and *∑* is 1.0 for both options. However, even though the contrast between the terminal probabilities of reward is indeed null, the delay-based task introduces a new source of contrast, that between the reward immediacies experienced with each terminal stimulus of each option. To illustrate, suppose that the short delay is 5 s and the long delay is 20 s. Then the ∆ of immediacies in the informative option would be 0.15 = [(1/5) − (1/20)] and the ∆ of immediacies in the non-informative option would be 0 because the average time to food is 12.5 s in the presence of both S^1^ and S^2^.

Thus, given this new source of contrast and given that *∑* remains the same for both options, Eq. 2 reduces to a one-parameter equation,

3 $${P}_{{\text{info}}}=\frac{1}{1+{e}^{-\beta \left(\frac{1}{{d}_{s}}-\frac{1}{{d}_{l}}\right)}}$$

where *d*_*s*_ and *d*_l_ correspond to the short and long delays, respectively. If we let *d*_l_/*d*_*S*_ = *r* and *d*_*s*_ + *d*_*l*_ = *S*, Eq. 3 becomes

4 $${P}_{{\text{info}}}=\frac{1}{1+{e}^{-\frac{\beta }{S}\left(r-\frac{1}{r}\right)}}$$

**Fig. 2:**
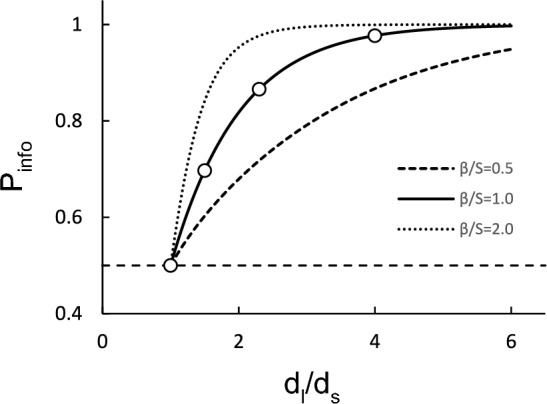
Predictions of the modified *Δ*–*Σ* model. Predictions of the modified *Δ*–*Σ* model when *β/S* = 0.5 (dashed curve), *β/S* = 1 (solid curve), and *β/S* = 2 (dotted curve). The white dots show the predictions for the current experimental conditions. The dashed horizontal line corresponds to indifference

Figure 2 shows preference for the informative option as predicted by Eq. 4 when *β/S* = 0.5, 1.0, and 2.0. Two predictions are noteworthy: (1) When the two delays are equal (*d*_*l*_/*d*_*S*_ = 1), the model predicts strict indifference between the two options regardless of *β/S*, and (2) as the delays become more dissimilar (i.e., as *d*_*l*_/*d*_*s*_ increases as the sum remains constant), preference for the informative option should increase in a logistic-like way modulated by the specific *β/S*.

A functional ecologically-based account allows for similar predictions. Because, under natural circumstances, information typically comes with instrumental value, we hypothesize that a bias for information may have been sculpted by natural selection across generations. After all, foraging animals continuously face cues associated with different and often uncertain delays to food. Being able to anticipate the time to food in such a temporally fluctuating environment may afford an instrumental advantage that could be used to exploit the often time-limited foraging opportunities available. For example, an animal may be willing to accept a ‘short’ delay to capture a given prey but reject the opportunity to pursue a prey when the expected delay exceeds a given threshold of acceptability. By using this decision rule, the animal would concentrate efforts on the most profitable prey and thus improve the overall rate of return. Thus, if this information bias is indeed built-in, one can anticipate a preference for signaled over unsigned delays. Furthermore, one can also argue that such a preference should be modulated by how different the delays are. To exemplify, imagine a forager facing an option yielding food, sometimes after a short delay, sometimes after a long one. When the two possible delays are considerably different (e.g., 5 s and 20 s) compared to similar (e.g., 10 s and 15 s), information regarding the length of the current delay should be more valuable because the benefits of rejecting long delays would have a larger impact on the average rate of capture. In other words, because the putative value of information should increase as the possible delays become more dissimilar, so should the preference for signaled delays.

Some have also argued that information is reinforcing per se. The claim of what could be labeled as the *intrinsic value hypothesis* is that animals attach inherent value to information about future outcomes independently of any instrumental purpose (e.g., Bennett et al. 2016; Eliaz and Schotter 2007; Grant et al. 1998; Kreps and Porteus 1978). In fact, some findings suggest that the value of non-instrumental information shares with the value of primary reward a common neural code (Bromberg-Martin and Hikosaka 2009; Kobayashi and Hsu 2019). Nonetheless, the question of why and how information became intrinsically valuable remains unanswered. Most important, this proposal predicts a preference for signaled delays but not its modulation by the similarity between possible delays because the value of information is not driven by any instrumental edge.

The evidence to date suggests that animals may prefer signaled to unsignaled delays, at least when the possible delays are very different. Bower et al. (1966) used a procedure similar to the one depicted in the bottom panel of Fig. 1 and found a preference for signaled delays when the long delay was four times longer than the short delay. Frankel and Vom Saal (1976) reported even stronger preferences with a threefold difference between delays. Similar findings have also been reported as a preference for multiple over mixed schedules of reinforcement (e.g., Alsop and Davison 1986; Davison 1972; Fantino 1969; Hursh and Fantino 1974; Richards 1981).

To our knowledge, the effect of the similarity between delays has not been previously studied. Both the modified *Δ*–*Σ* hypothesis and an ecologically-driven analysis suggest that preference for signaled delays should be modulated by the similarity between delays—the more dissimilar the delays, the stronger the preference. On the other hand, the intrinsic value hypothesis predicts no modulation of preference by delay similarity. The experiment that follows puts the issue to the test. The procedure followed closely the one depicted in the bottom panel of Fig. 1 with options differing only in that one had distinctive cues signaling the short and the long delays, while the other had ambiguous cues (short and long delays were equally likely in their presence). Across conditions, we varied how different the delays were (*r* = *d*_*l*_/*d*_*s*_) while keeping their total duration (*S* = *d*_*s*_ + *d*_*l*_) constant.

## Method

### Subjects

Seven pigeons (*Columba livia*), between 80 and 85% of their free-feeding weights, were used in this experiment. They were individually housed in a temperature-controlled room (around 21 °C) on a 13:11 h light/dark cycle (lights on at 8:00). Pigeons had previous experience with the suboptimal choice procedure described in Fortes et al. (2016), including the stimuli used in this experiment. We implemented a corrective procedure during *Pretraining* (see below) to reduce potential biases. Grit and water were always available in the home cage. The pigeons were cared for according to the animal care guidelines of the Directorate-General for Food and Veterinary (DGAV), the Portuguese national authority for animal health, and the University of Minho. All experimental procedures were conducted in agreement with European (Directive 2010/63/EU) and Portuguese law (Ordinance 1005/92 of October 23), and were approved by DGAV (Authorization #024946).

### Apparatus

Three Med Associates operant boxes for pigeons were used. The boxes were 28.5-cm high, 24-cm long, and 30-cm wide. Each box was enclosed in a sound-attenuating chamber, equipped with a fan that circulated air and masked extraneous noises. The response panel had three circular keys, each 2.5 cm in diameter, and placed 6 cm apart (center-to-center), with the lowest edge 21 cm above the floor grid. The response panel also included a 6-cm wide × 5-cm high opening, centered 4 cm above the floor grid. The pigeon had access to food when the opening was illuminated by a 1.1-W light and the food hopper was raised. In the panel opposite to the response panel, a houselight (2.8 W) centrally located 23 cm above the floor illuminated the whole box. A personal computer with ABET II software (Lafayette Instruments) controlled the events and recorded data. Communications with the experimental chambers used the Whisker interface (Cardinal and Aitken 2010).

### Procedure

*Pretraining*. Pigeons were initially trained on different Fixed-Ratio (FR) schedules to reduce any carryover effects from their previous experiments. Each color (red, green, yellow, and blue) and symbol (cross and circle) was presented eight and four times per session, respectively. Color stimuli were always shown on the center key and the symbol stimuli were shown equally often on each side key. Once the FR schedule was completed, the pecked key was turned off and the feeder was raised for 3–5 s adjusted for each pigeon to maintain its body weight. After food, a 10 s Inter-Trial Interval (ITI) followed with the house light on. Pigeons were trained for two sessions with a FR1 schedule and for one further session with a FR5 schedule in effect during the first half of the session and a FR10 in effect during the second half.

#### Experimental task

After pretraining, pigeons were exposed to the procedure depicted in the bottom panel of Fig. 1. Each session comprised 96 trials, 32 choice trials, and 64 forced trials. During the initial link of a choice trial, the left and right keys were illuminated with symbols (Cross and Circle). A single peck on either side key turned off both keys and gave way to the corresponding terminal stimuli. The terminal delay to food in both alternatives was either short or long, each occurring on 50% of the trials. When the informative option was chosen, short delays elapsed with the center key illuminated with one color stimulus (*S*^*S*^), whereas long delays elapsed with the center key illuminated with a different color (*S*^*L*^). When the non-informative option was chosen, short and long delays to food elapsed with the center key illuminated with one of two stimuli (*S*^*1*^ or *S*^*2*^), each occurring randomly on 50% of the occasions. Forced trials had the same structure as choice trials, except that only one of the alternatives was presented at trial onset. They were pseudo-randomly distributed such that of the 32 trials with each option, 16 occurred on the left-side key and 16 on the right-side key. Choice trials were also pseudo-randomly arranged such that the left vs. right location of the options was balanced. The initial-link symbols and the terminal-links colors were counterbalanced across pigeons. The time between the presentation of one (forced trials) or two (choice trials) options and the peck at one of the side keys defined the latency to respond on each trial.

Each pigeon went through two baseline and three experimental conditions differing in the long to short delay ratio. In baseline, all the delays to food were set at 12.5 s (long*/S*hort ratio = 1.0). In the other conditions, the short and long delays became increasingly different, but the average delay remained at 12.5 s. The delays used were 10 and 15 s (long*/S*hort ratio = 1.5), 7.5 and 17.5 s (long*/S*hort ratio = 2.3), and 5 and 20 s (long*/S*hort ratio = 4.0). All pigeons started and finished with the 1.0 ratio (baseline), with the order of the remaining conditions counterbalanced across pigeons. Table 1 shows the order experienced by each pigeon. Each condition lasted for a minimum of 10 sessions and remained in effect until stability was reached. Stability was assumed when during the last three sessions (a) there was no strictly increasing or strictly decreasing trend in preference and (b) the range of choice proportions was at most 15%.

**Table 1:** Ratio of long*/s*hort duration (number of sessions) for each pigeon and condition

Pigeon	Order of conditions				
	1	2	3	4	5
212	1.0 (16)	4.0 (16)	2.3 (17)	1.5 (15)	1.0 (15)
283	1.0 (15)	1.5 (17)	2.3 (15)	4.0 (15)	1.0 (17)
456	1.0 (15)	1.5 (16)	4.0 (15)	2.3 (15)	1.0 (25)
595	1.0 (15)	2.3 (16)	1.5 (15)	4.0 (17)	1.0 (15)
860	1.0 (16)	4.0 (15)	2.3 (15)	1.5 (16)	1.0 (18)
916	1.0 (16)	2.3 (15)	1.5 (16)	4.0 (15)	1.0 (18)
1727	1.0 (15)	1.5 (15)	4.0 (16)	2.3 (16)	1.0 (16)

1.0 (short = long = 12.5 s), 1.5 (short = 10 s; long = 15 s), 2.3 (short = 7.5 s; long = 17.5 s), 4.0 (short = 5 s; long = 20 s). The number of sessions per condition is shown in parentheses

#### Data analysis

Our primary dependent measures were the proportion of choices for the informative option, the latencies to respond to each option, and the response rate during the four terminal stimuli, all during the last three sessions of each condition. Prior to analysis, latency data were successfully normalized using a natural log transformation. Violations of sphericity were corrected by the Greenhouse–Geisser method. A Type-1 error rate of 0.05 was adopted for all statistical comparisons.

## Results

On average, pigeons took 16 sessions to complete each long*/*short ratio (range 15–25). Table 1 shows the number of sessions per ratio for each bird. A repeated-measures ANOVA revealed that the amount of training to reach stability did not vary significantly across conditions [*F*(4,24) = 2.09, *p* = 0.114].

**Fig. 3:**
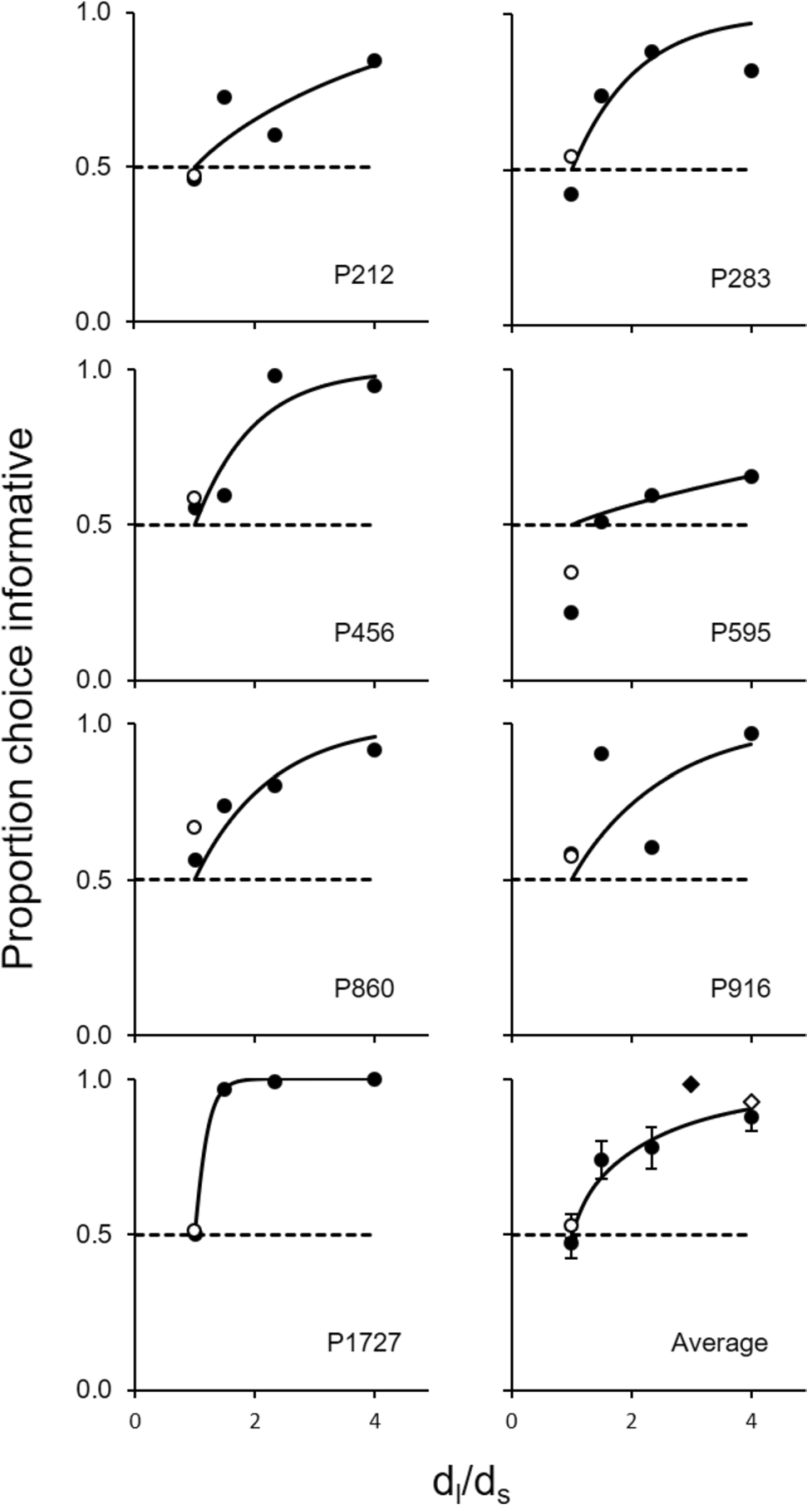
Proportion of choices for the informative option. Note. Proportion of choices for the informative option as a function of the delay ratio (dots). The empty dot corresponds to the second baseline condition. The solid lines show the best-fitting predictions of the modified version of the *Δ*–*Σ* hypothesis. The dashed horizontal lines correspond to indifference. Bottom right panel: The solid line is the average of the individual fits. The error bars represent ± 1 standard error of the mean (SEM). The filled and unfilled diamonds show the average preference reported by Frankel and Vom Sall (1976) and Bower et al. (1966), respectively

The symbols in Fig. 3 show preference for the informative option as a function of the delay ratio for each subject. The lower right panel shows the average preference across birds. As expected, pigeons were indifferent between the options when the long*/*short ratio = 1.0, both in the first and second baseline conditions (filled and unfilled dots, respectively): the average preferences for the informative option (± SEM) were 0.47 (± 0.047) and 0.53 (± 0.038), respectively. However, with the 1.5, 2.3, and 4.0 long*/S*hort ratio, the average preference increased to 0.74 (± 0.061), 0.78 (± 0.068), and 0.88 (± 0.044), respectively. These preferences did not differ significantly from chance when the long*/S*hort ratio = 1.0 [largest *t*(6) = 0.70, *p* = 0.513], but were significantly above chance in the remaining long*/S*hort ratios [smallest *t*(6) = 3.97, *p* = 0.007, *d* = 1.498]. Despite some variability between and within pigeons, preference for the informative option increased with the long*/S*hort ratio. A repeated-measures ANOVA confirmed that this trend was statistically significant [*F*(3,18) = 16.51, *p* < 0.001, *η*^2^ = 0.733].

The solid lines on each bird’s panel of Fig. 3 show the best-fitting predictions of the modified *Δ*–*Σ* hypothesis obtained by the least-squares method. The best-fitting parameter, β, for each pigeon is shown in Table 2. The solid line in the average panel of Fig. 3 (bottom right) shows the average of the individual fits. Overall, the model captures well the general increasing trends and the average fit accurately describes the average preference.

**Table 2:** Best fitting parameter and R^2^ for the modified *Δ*–*Σ* hypothesis

Pigeon	*β/S*	*R*^*2*^
212	0.421	0.858
283	0.949	0.908
456	1.028	0.917
595	0.173	0.884
860	0.830	0.955
916	0.702	0.644
1727	4.109	0.999

**Fig. 4:**
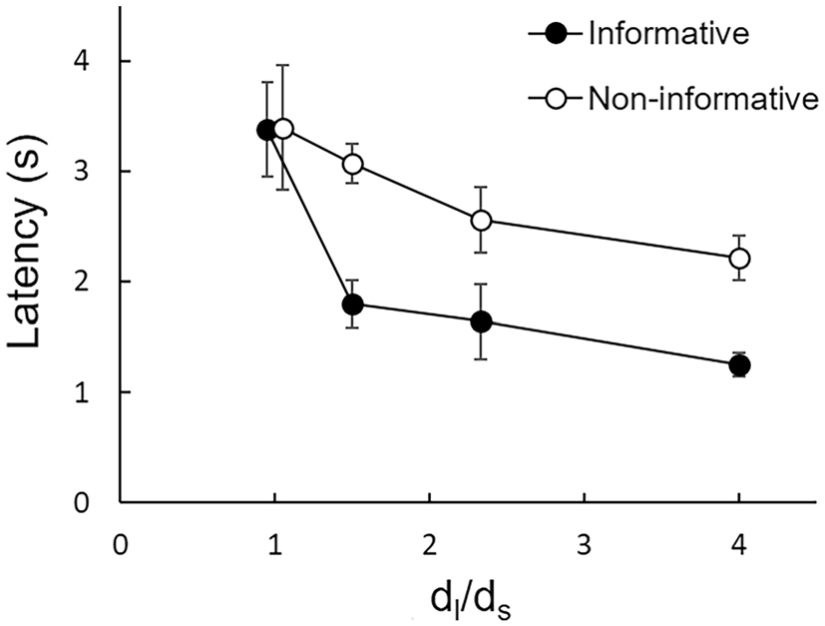
Latencies to accept each option for each long*/*short ratio. Average median latencies to respond to the informative (filled symbols) and the non-informative (unfilled symbols) options in forced trials during the last three sessions of each long*/S*hort ratio. Baseline latencies are laterally displaced for visualization purposes. The error bars show ± 1 SEM

Previous research has shown that latencies to respond can be used as a sensitive metric of value and preference: Organisms usually respond faster to preferred than to non-preferred alternatives when presented individually (e.g., Aw et al. 2012; Lagorio and Hackenberg 2012; Macías et al. 2021; Monteiro et al. 2020; Reboreda and Kacelnik 1991; Shull et al. 1990; Vasconcelos et al. 2013; for reviews see Kacelnik et al. 2011, 2023). We analyzed the latencies to respond to the initial link during forced trials in each condition in search of converging evidence regarding the effect of the long*/*short ratio on preference. As expected from choice data, latencies to accept each option were similar in both baselines (long*/S*hort ratio = 1.0) and were thus averaged into a single condition [largest *t*(6) = 1.58, *p* = 0.191]. Figure 4 shows the average of the median latencies to respond to each option on each long*/S*hort ratio. As the long*/S*hort ratio increased, latencies to accept the options diverged, with animals expressing longer latencies to accept the non-preferred, non-informative option than to accept the preferred, informative option. These visual impressions were confirmed by a two-way repeated-measures ANOVA with option (informative vs. non-informative) and the long*/S*hort ratio as factors ran on log median latencies. Both the main effects of option and ratio were significant [*F*(1,6) = 11.09, *p* = 0.016, *η*_p_^2^ = 0.649; Greenhouse–Geisser corrected *F*(1.78,10.66) = 10.53, *p* = 0.004, *η*_p_^2^ = 0.637, respectively], as well as their interaction [*F*(3,18) = 7.02, *p* = 0.003, *η*_p_^2^ = 0.539]. The latter indicates that latencies to accept each option progressed differently as the *d*_*l*_/*d*_*s*_ ratio increased: while latencies to accept the non-informative option remained roughly constant (the decrease is non-significant), latencies to accept the informative option decreased significantly as expected because the larger the ratio the more attractive this option becomes. Overall, the observed differences in latencies to accept each option are consistent with the preference data.

Lastly, we looked at response rates at the four terminal stimuli (*S*^*S*^, *S*^*L*^, *S*^*1*^, and *S*^*2*^). Four of the seven pigeons did not peck at any of the terminal stimuli (an average of fewer than 2 pecks per trial across the whole delay). For the remaining three pigeons, when the delays were unequal (*d*_*l*_/*d*_*s*_ > 1), response rate at *S*^*S*^ was consistently higher than at *S*^*L*^. The response rates to *S*^*1*^ and *S*^*2*^ were roughly similar and, when averaged, tended to fall between the rates for *S*^*S*^ and *S*^*L*^. These findings suggest that these pigeons learned the delay (or mixture of delays) signaled by each terminal stimulus and modulated their response rate accordingly.

## Discussion

This experiment analyzed the effect of signaling different delays to food on choice. Pigeons chose between two alternatives, each leading to food after an equally likely short or long delay. The alternatives differed in that, for one of them, the short and long delays were associated with distinctive cues (informative option) and, for the other one, the delays were not associated with distinctive cues (non-informative option). We found that pigeons reliably prefer the informative alternative when the signaled delays differed, even though both alternatives led to the same overall rate of reward. An examination of response latencies as a measure of value buttressed our interpretation as pigeons expressed significantly shorter latencies to respond at the informative than at the non-informative option when the long*/*short ratio > 1.0. An analysis of the response rates at the terminal stimuli, although limited to three subjects, confirmed that the cues allowed pigeons to identify the ongoing delay in the informative but not in the non-informative option. Importantly, we also found that the preference for signaled delays increases in an orderly fashion with the ratio of the delays.

These findings are consistent with those reported by Bower et al. (1966) and Frankel and Vom Saal (1976). The bottom right panel of Fig. 3 shows their average findings. Using a long*/S*hort ratio of 4 (FI 40 vs. FI 10), Bower et al. (1966) reported an average preference very similar to the one we found (cf. unfilled diamond). Frankel and Vom Saal, on the other hand, reported an even stronger preference for the signaled option (cf. filled diamond) with a smaller long*/S*hort ratio of 3 (FI 45 vs. FI 15). The reasons for this somewhat higher preference relative to the ones reported here and by Bower et al. remain unclear. Perhaps the contrast between the successful manipulation and the previous five conditions where no preference emerged inflated preference to values that would not have been observed otherwise. This reasoning is nonetheless speculative and complicated by cross-experiment comparisons.

The observed modulation of preference by the long*/S*hort ratio is inconsistent with the intrinsic value hypothesis (cf. Bennett et al. 2016; Eliaz and Schotter 2007; Grant et al. 1998; Kreps and Porteus 1978). Were that the case, one would expect similar preferences for the informative option whenever the long/ratio > 1. The increase in preference for the informative option as the long*/S*hort ratio increased suggests, on the contrary, that the value of information about future outcomes is not *intrinsic*; it depends on the potential instrumental advantage it conveys.

On the other hand, our findings are consistent with the predictions of the modified *Δ*–*Σ* hypothesis and the ecologically-based account. Regarding the *Δ*–*Σ* hypothesis, despite some variability within and between subjects, the average of the individual fits captured well our average findings (cf. bottom right panel of Fig. 3). The new source of contrast—that between the immediacy of reward experienced with each terminal stimulus within each option—proved pivotal both in the design of the experiment and the interpretation of results. Our findings thus buttress the model’s assumption that, all else equal, the value of an alternative varies directly with the *Δ* of immediacies: Greater ratios between the two terminal stimuli immediacies, for the same delay average, mean greater value of the alternative (see Fig. 2). The model also predicts that, for a constant ratio, preference for the informative option should decrease with the average (or sum), a novel prediction that remains to be tested. Furthermore, our results encourage the extension of the model to other dimensions of reward besides probability and delay, dimensions that may be additional sources of contrast.

Ecologically, an increased preference for signaled delays as the long*/S*hort ratio increases tracks the putative increase in information utility—the benefit of avoiding long delays increases with larger long*/S*hort ratios. Under natural circumstances, animals can use information to adjust their behavior and thus abandon patches when the rate of return falls below some threshold. In practice, when foraging, an animal can pursue a prey if the available cues signal a short waiting time for food or continue searching if the available cues signal delays beyond a threshold of acceptability (e.g., Charnov 1976; Parker and Stuart 1976; for a review, see Vasconcelos et al. 2017). In the present study, pigeons learned the contingencies associated with each stimulus, used the information so conveyed to guide preference, but did not collect the benefit of avoiding long delays: they had to endure all the waiting times in both alternatives (i.e., pay the opportunity cost). In other words, even though the foraging mechanisms may have been sculpted to gather and use information, the information gathered in the artificial laboratory preparation cannot be used—the domain of selection mismatched the domain of testing (Fortes et al. 2016; Stevens and Stephens 2010). Thus, pigeons behaved in the experimental preparation as if information were usable; they seemingly deployed decision mechanisms evolved to deal with the statistical properties of natural environments, not with the artificial preparations of unavoidable opportunity costs.

More generally, a preference for options that signal what event will occur and when may impart somewhat unappreciated benefits, particularly in the form of adaptive anticipatory responses. Evidence for this claim has been accumulating in a variety of domains, including aggressive behavior (e.g., Hollis 1984, 1990; Hollis et al. 1995), aversive conditioning (e.g., Blustein et al. 1997; Fanselow and Baackes 1982; Mongeluzi et al. 1996), drug tolerance (e.g., Grisel et al. 1994; Larson and Siegel 1998; Siegel 1975; Siegel et al. 2000), feeding and digestion (e.g., Woods 1991; Woods and Ramsay 2000; Woods and Strubbe 1994; Zamble 1973), maternal behavior (e.g., Tancin et al. 2001), and sexual behavior (e.g., Domjan et al. 1986; Hollis et al. 1989, 1997; Zamble et al. 1985), among others. In a particularly illustrative example, Hollis et al. (1997) allowed male blue gourami to copulate and tend eggs either after being exposed to a sexually conditioned stimulus or without such prior exposure. Males with signaled sexual encounters showed reduced aggressive behavior toward the female, shorter spawning latencies, and increased nest-building behavior, among others. Notably, compared to unsignaled encounters, the signaled sexual encounters resulted in more than 10 times as many offspring.

We argue that the preference for informative options or a general bias for information is consistent with the premise that the function of learning mechanisms is to enhance the organisms’ interaction with biologically relevant events rather than the acquisition of arbitrary relations (e.gDomjan 2005; Hollis 1997; Shettleworth 1994) as sometimes the excessive focus on responses to antecedents of biologically relevant events suggests.

To conclude, in this experiment, we showed that the preference for informative over non-informative options extends naturally to a situation where information is about delays, not probabilities of reward. We found that preference for signaled delays varied with the ratio between the long and short delays within alternatives. From a normative standpoint, this preference and trend may occur because animals use evolved mechanisms shaped to deal with usable information: they use information to maximize the rate of food intake by avoiding, for example, relatively delayed opportunities (in comparison with the background) and by deploying anticipatory responses that improve their interaction with biologically relevant events. Although this strategy cannot be implemented in the experimental condition, we believe it reflects the conditions where it evolved and where most, if not all, information can be instrumentally used. Although, this line of reasoning permits a better understanding of selective pressures shaping behavior and derive optimal policies, it cannot pinpoint the specific behavior-generator. To that end, we resorted to a modified version of he *Δ*–*Σ* hypothesis incorporating a new type of contrast: that between reward immediacies. The modified version accounted well for our data. Together, these approaches permit a first glimpse at the ultimate and proximate causes of preference for signaled delays.

## Data Availability

On request.
